# Enhanced NO_2_ Gas Sensing Properties Based on Rb-Doped ZnO/In_2_O_3_ Heterojunctions at Room Temperature: A Combined DFT and Experimental Study

**DOI:** 10.3390/s24165311

**Published:** 2024-08-16

**Authors:** Yaning Yang, Jiawen Cui, Zhihua Luo, Zhixin Luo, Yanhui Sun

**Affiliations:** 1 College of Information &Communication Engineering, Dalian Minzu University, Dalian 116600, China; cjw@dlnu.edu.cn (J.C.); luo_zhihua8@163.com (Z.L.); lzx@dlnu.edu.cn (Z.L.); syh@dlnu.edu.cn (Y.S.); 2 School of Mechanical Engineering, Dalian University of Technology, Dalian 116024, China

**Keywords:** NO_2_ gas sensor, ZnO, In_2_O_3_, heterojunctions, DFT

## Abstract

In this work, alkali metal Rb-loaded ZnO/In_2_O_3_ heterojunctions were synthesized using a combination of hydrothermal and impregnation methods. The morphology and structure of the synthesized samples were characterized by X-ray diffraction, field emission scanning electron microscopy, and transmission electron microscopy. The enhancement mechanism of the nitrogen dioxide gas sensing performance of the Rb-loaded ZnO/In_2_O_3_ heterojunctions was systematically investigated at room temperature using density-functional theory calculations and experimental validation. The experimental tests showed that the Rb-loaded ZnO/In_2_O_3_ sensor achieved an excellent response value of 24.2 for 1 ppm NO_2_, with response and recovery times of 55 and 21 s, respectively. This result is 20 times higher than that of pure ZnO sensors and two times higher than that of ZnO/In_2_O_3_ sensors, indicating that the Rb-loaded ZnO/In_2_O_3_ sensor has a more pronounced enhancement in performance for NO_2_. This study not only revealed the mechanism by which Rb loading affects the electronic structure and gas molecule adsorption behavior on the surface of ZnO/In_2_O_3_ heterojunctions but also provides theoretical guidance and technical support for the development of high-performance room-temperature NO_2_ sensors.

## 1. Introduction

Nitrogen dioxide (NO_2_) is a toxic and corrosive gas with an irritating odor. NO_2_ not only has a significant negative impact on human health, but also has a significant damaging effect on the quality of the environment [1]. NO_2_ is one of the major pollutants in industrial emissions and traffic exhausts and is strongly toxic and oxidizing [2]. The Health and Safety Rule Alert states that human beings should avoid spending more than 8 h in an environment with NO_2_ at 1 ppm; otherwise, it can cause cardiovascular diseases [3]. Especially prolonged exposure to low concentrations of NO_2_ (10 ppm) can lead to bronchitis and even death [4]. In addition, NO_2_ can generate ozone (O_3_) and acid rain through chemical reactions in the atmosphere, further harming the environment and ecosystem [5].

In recent years, semiconductor oxide-based gas sensors have received significant attention due to their simple structure, high sensitivity, and low cost. Among them, SnO_2_ [6], ZnO [7], In_2_O_3_ [8], and WO_3_ [9], as typical n-type semiconductor materials, are suitable materials for NO_2_ sensor research due to their excellent electron transport properties and chemical stability. However, single materials often suffer from slow response speeds, poor selectivity, and high operating temperatures in practical applications. In order to overcome these drawbacks, researchers have begun to combine different materials to form heterojunctions, which can significantly improve the sensing performance. Among many heterojunction materials, ZnO/In_2_O_3_ [10] heterojunctions show excellent gas-sensing performance due to their good energy band matching and interfacial charge transfer properties. For example, Yuan et al. [11] prepared ZnO/In_2_O_3_ heterostructured nanosheets using a one-step hydrothermal method and discussed the effect of ZnO content on n-butanol gas-sensing performance. Liang et al. [12] achieved rapid detection of ppb-level NO gas in ZnO/In_2_O_3_ nanocomposites at room temperature (RT) using resonant tunneling modulation. Huang et al. [13] synthesized a one-dimensional ZnO/In_2_O_3_ nanofiber sensor based on a coaxial electrospinning method, which demonstrated superior selectivity and stability for ethanol gas.

Functionalization with noble [14,15], transition [16], and alkaline metals [17] has also emerged as an effective method to improve sensing performance. It has been reported that loaded metals can act as catalysts to promote the chemical reactions of target gas molecules and improve the response speed and sensitivity of sensors. Meanwhile, metal loading can change the electronic structure of semiconductor materials and regulate their surface electron density, thus affecting the conductivity change of the sensor. For example, Liu et al. [18] loaded Ag onto ZnO/In_2_O_3_ nanofibers, demonstrating excellent sensitivity, superior selectivity, and long-lasting stability for formaldehyde gas. Wang et al. [19] realized Au-decorated ZnO/In_2_O_3_ belt-tooth-shaped nanoheterostructures using a chemical vapor deposition process. The gas sensor exhibited excellent sensing performance for C_2_H_2_ gas at low operating temperatures. Guo et al. [20] designed ultra-small Pt nanoparticle-functionalized ZnO/In_2_O_3_ nanofibers using a new catalyst-loaded platform (Pt@ZIF-8). The experimental results showed that the Pt-ZnO/In_2_O_3_ nanofiber-based sensor has excellent acetone response, ultrafast response and recovery time, and a low detection limit.

Although several studies have focused on the application of loaded metals and ZnO/In_2_O_3_ [21] heterojunctions for detecting NO_2_ gas, the mechanism by which Rb loading enhances the NO_2_-sensing performance of ZnO/In_2_O_3_ heterojunctions at room temperature has not yet been systematically investigated. Room-temperature detection can reduce energy consumption, making the sensor more economical and environmentally friendly in practical applications. In addition, NO_2_ sensors under room-temperature conditions can realize a wider range of applications, including indoor air quality monitoring, portable environmental testing equipment, and mobile monitoring stations, which all place high demands on the power consumption and stability of the sensors. Therefore, it is of great practical importance to develop NO_2_ sensors that can operate efficiently at room temperature.

The aim of this study was to investigate, for the first time, the synergistic effect of Rb-loaded ZnO/In_2_O_3_ heterojunctions in room-temperature NO_2_ sensing using a combination of density functional theory (DFT) calculations and experiments. The Rb-loaded ZnO/In_2_O_3_ heterojunctions were prepared using hydrothermal and impregnation methods. The morphological structures and surface chemical states were observed using X-ray diffraction (XRD), transmission electron microscopy (TEM), and X-ray photoelectron spectroscopy (XPS). Through theoretical calculations and experimental validation, the mechanism by which loaded Rb influences the electronic structure of the ZnO/In_2_O_3_ heterojunction surface and the adsorption behavior of gas molecules was revealed. This is expected to provide new ideas for the development of high-performance, room-temperature NO_2_ sensors, as well as theoretical guidance and technical support for future sensor design.

## 2. Materials and Methods

### 2.1. Preparation of ZnO and In_2_O_3_ Heterojunctions

Zinc nitrate (Zn(NO_3_)_2_·6H_2_O), indium nitrate (In(NO_3_)_3_·XH_2_O), and sodium hydroxide (NaOH) were purchased from Tianjin Kemiou Chemical Reagent Co., Ltd. (Tianjin, China). Zinc nitrate and indium nitrate with a stoichiometric ratio of 2:1 (0.6:0.3 g) were dissolved in 20 mL of deionized water and stirred at 80 °C for 15 min. Subsequently, they were transferred to a 50 mL pipette, and the solution was ultrasonically pulverized for 10 min while 10 mL of 2 mol/L sodium hydroxide solution was rapidly injected. Then, it was cross-washed with deionized water and ethanol three times and air-dried at 60 °C. Finally, the dried samples were placed in a tube furnace and heated to 850 °C at a heating rate of 5 °C/min and then calcined for 2 h. ZnO/In_2_O_3_ heterojunction nanocomplexes were obtained.

### 2.2. Preparation of Rb-Loaded ZnO/In_2_O_3_ Heterojunctions

Rubidium nitrate (RbNO_3_) was purchased from Shanghai Maclin Biochemical Technology Co., Ltd. (Tianjin, China). First, 0.166 g of the ZnO/In_2_O_3_ heterojunction complex was added to 5 mL of deionized water and stirred for half an hour. Then, 0.147 g of rubidium nitrate was added to 5 mL of ethanol and stirred for half an hour. The above solutions were mixed and ultrasonicated for 60 min before being vacuum-dried at 70 °C for 24 h. Then, the powder was pyrolyzed in an argon atmosphere at 500 °C for 30 min to obtain 1 mol% Rb-ZnO/In_2_O_3_ [22]. Finally, 2% Rb-ZnO/In_2_O_3_ and 3% Rb-ZnO/In_2_O_3_ were prepared through adding 0.294 and 0.441 g of rubidium nitrate, respectively, with all other conditions held constant.

### 2.3. Material Characterization

The samples’ crystal structure and composition were determined using an X-ray diffractometer (XRD, D/Max 2400, Rigaku, Tokyo, Japan) in the 2θ region of 10°–80° with Cu Kα radiation at a rate of 3°/min. The morphology of the samples was obtained using a field emission scanning electron microscope (SEM; Apreo 2C, Thermo Scientific, Waltham, MA, USA). The internal nanostructure and elemental distribution of the samples were characterized using transmission electron microscopy (TEM; Talos F200S, Thermo Scientific, Waltham, MA, USA) at 200 kV. The composition and chemical states of the elements in the materials were analyzed using a Thermo Fisher K-ALPHA X-ray photoelectron spectrometer (XPS; Thermo Fisher, Waltham, MA, USA). The charge correction was performed with the C 1s peak (284.8 eV) as a reference.

### 2.4. Fabrication of Sensors and Measurement

A 2 mg sample was mixed with 25 µL of deionized water and thoroughly ground to form a slurry. This slurry was uniformly applied to an alumina substrate pre-coated with gold electrodes (5 × 10 mm). The sensor was dried at room temperature for 12 h to ensure complete evaporation of the water. A cross-sectional image of the sensor was observed using scanning electron microscopy (SEM), as shown in Figure S1. It can be seen that the sensing material formed a uniform sensing film on the substrate, with a thickness of approximately 65 µm.

**Figure 1:**
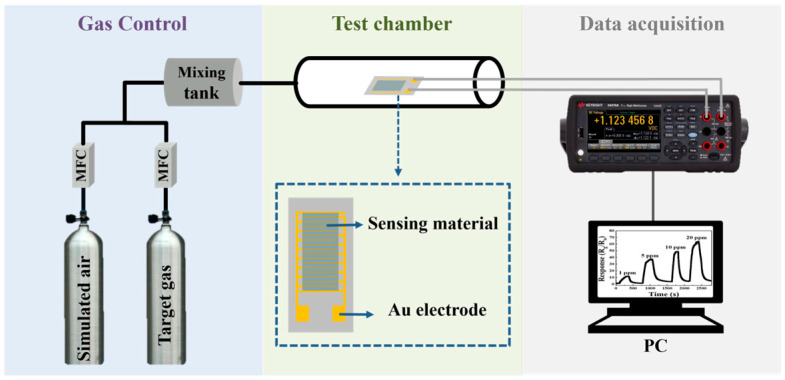
Schematic diagram of the gas sensor performance test.

The sensor’s performance was evaluated using a dynamic gas flow device, as shown in Figure 1. All gases used in this study were supplied in certified cylinders by Dalian GuangMing Special Gas Products Co., Ltd. (Dalian, China). The composition of the simulated air was 70% N_2_ and 30% O_2_. All other gases were at a concentration of 100 ppm. The simulated air and target gases were mixed into different concentrations in a mixing tank under the precise control of a mass flow controller (MFC; Criterion D500, Horina, Kyoto, Japan) and introduced into the test chamber at a steady flow rate of 300 sccm. The sensors were placed in a test chamber for electrical measurement, with resistance values recorded by a source meter (34470A, Keysight, Santa Rosa, CA, USA) and a computer-aided measurement instrument.

The gas response value was defined as S = R_a_/R_g_ (ammonia, acetone, methanol, formaldehyde, and ethanol) or R_g_/R_a_ (NO_2_), where R_a_ and R_g_ are the resistance values of the sensor exposure to air and target gas, respectively. The error on the response value was calculated by applying error propagation, considering the load resistance tolerance and instrument uncertainty on the output voltage acquisition [23]. The sensor response and recovery times were determined through measuring the time taken for the sensor to reach 90% of the saturation resistance value during adsorption and desorption [24].

Sensitivity: The sensor was exposed to increasing concentrations ranging from 1 to 20 ppm of NO_2_ to sound out different application scenarios.

Repeatability: A 2 mol% Rb ZnO/In_2_O_3_ film was exposed to five cycles of 10 ppm of NO_2_. 

Selectivity: The concentrations of the selected gases (NO_2_, ethanol, formaldehyde, methanol, acetone, and ammonia) were determined on the basis of the World Health Organization’s annual publication (Air Quality Guidelines) [25], as well as average test levels reported in the literature.

The limit of detection (LOD) of the sensor can be calculated using the following Equation (1) [6].

LOD = 3σ/s(1)

where σ is the standard deviation of the response value obtained from the blank measurements, where 40 data points were tested in the absence of the target gas; s is the slope of the calibration curve.

### 2.5. Periodic DFT Calculation Details

**Figure 2:**
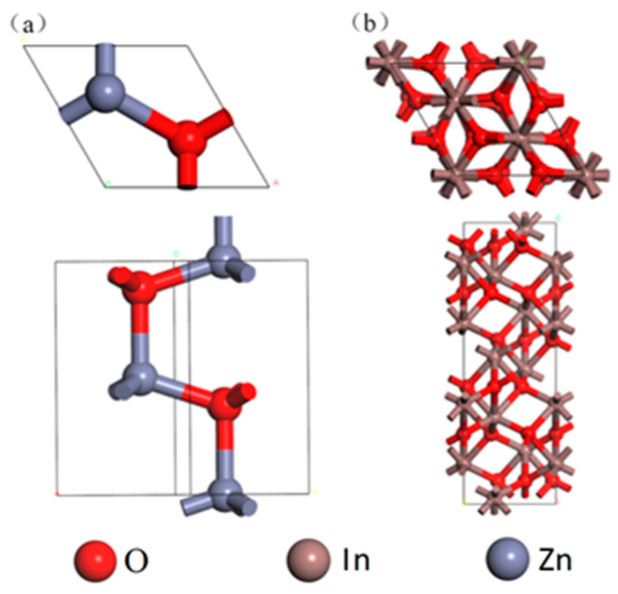
Single-cell structure of (**a**) ZnO and (**b**) In_2_O_3_.

In this work, density functional theory (DFT) calculations were conducted using the Vienna Ab initio Simulation Package (VASP 5.2) [26,27,28]. Generalized gradient approximation (GGA) was applied for the exchange-correlation energy and interatomic interactions with Perdew–Burke–Ernzerh adsorption configurations. The cut-off energy was set at 400 eV. Monkhorst–Pack k-point meshes (2 × 6 × 1) were employed for structural relaxation within the Brillouin zone. The convergence criteria for energy and force were 1 × 10^−4^ eV and 3 × 10^−2^ eV/Å, respectively. In this work, the original computational model was adopted with a ZnO fibrillar zincite structure and an In_2_O_3_ rhombic-centered hexahedral structure, as shown in Figure 2.

## 3. Results and Discussion

### 3.1. Characterization of the Samples

**Figure 3:**
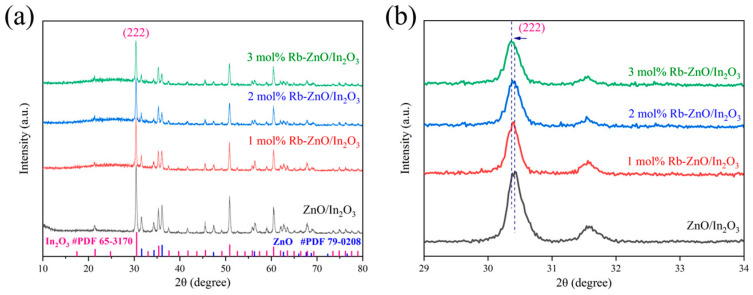
(**a**) XRD patterns and (**b**) high-resolution XRD patterns of 1–3 mol% Rb ZnO/In_2_O_3_ and ZnO/In_2_O_3_.

Figure 3 illustrates the XRD spectra of 1–3 mol% Rb-ZnO/In_2_O_3_ and ZnO/In_2_O_3_ composites. The XRD patterns of all four samples exhibited characteristic peaks of In_2_O_3_ and ZnO, as shown in Figure 3a. Among them, those diffraction peaks with 2θ values of 30.49°, 50.88°, and 60.49° belong to the crystalline facets of (222), (440), and (622) of In_2_O_3_ (JCPDS No. 65-3170), respectively. Meanwhile, those diffraction peaks with 2θ values of 31.60°, 33.30°, and 36.08° belong to the crystalline facets of (100), (002), and (101) of ZnO (JCPDS No. 79-0208), respectively. No other characteristic peaks were detected, indicating the high purity of the obtained composites. It is noteworthy that the loaded Rb metal element, due to its low content, resulted in the absence of characteristic diffraction peaks. This situation has been reported in several literature works [20]. High-resolution XRD maps of the four samples are shown in Figure 3b, in which the characteristic diffraction peaks of In_2_O_3_ (222) shifted as the loading of Rb increased. This is due to the fact that the ionic radius of Rb^+^ is larger than that of In^3+^ and Zn^2+^ [29,30,31,32].

**Figure 4:**
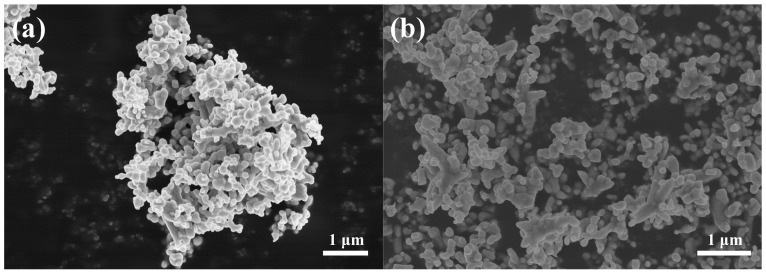
SEM images of (**a**) ZnO/In_2_O_3_ and (**b**) 2 mol% Rb-ZnO/In_2_O_3_.

Figure 4a shows an SEM image of ZnO/In_2_O_3_. The morphology consists of nanoparticles with diameters of approximately 400 and 100 nm, where the larger nanoparticles are ZnO and the smaller ones are In_2_O_3_. The SEM image is in general agreement with the images previously reported in the literature, which indicates that the sensitive materials prepared in this study are correct [12]. Figure 4b shows an SEM image of the ZnO/In_2_O_3_ composite with 2 mol% Rb loading. It can be seen that the loading of Rb did not destroy the overall structure of the ZnO/In_2_O_3_ heterojunction. The small particles of the In_2_O_3_ and Rb particles were dispersed around ZnO. Figure S2 shows SEM images of 1 and 3 mol% Rb-ZnO/In_2_O_3_, and it can be seen that several samples have similar morphology sizes, with only minor differences.

**Figure 5:**
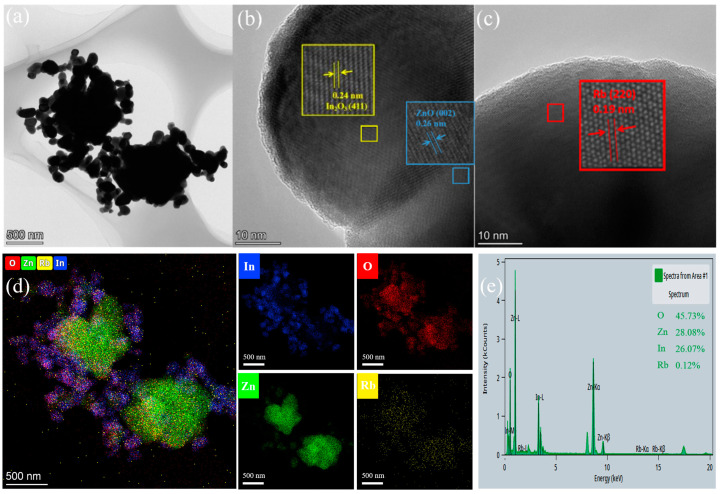
(**a**)TEM images of 2 mol% Rb-ZnO/In_2_O_3_, (**b**,**c**) HR-TEM images of a 2 mol% Rb-ZnO/In_2_O_3_ lattice, (**d**) corresponding EDS elemental mapping analysis of Zn (green), O (red), In (blue), and Rb (yellow), and (**e**) EDS counterpart of 2 mol% Rb-ZnO/In_2_O_3_ estimated elemental content.

Figure 5a shows a TEM image of 2 mol% Rb-ZnO/In_2_O_3_. The central part is a ZnO block approximately 800 nm in size, surrounded by small In_2_O_3_ particles approximately 50 nm in size and dispersed Rb particles. It can be seen that the 2 mol% Rb-ZnO/In_2_O_3_ composite maintains excellent distribution and size homogeneity. Figure 5b presents a HR-TEM image of 2 mol% Rb-ZnO/In_2_O_3_. It clearly shows the contact interface between two different crystal structures with lattice spacings of 0.24 and 0.26 nm, corresponding to the In_2_O_3_ (411) and ZnO (002) faces, respectively. Meanwhile, the lattice spacing of 0.19 nm belongs to the (220) crystal surface of Rb, as shown in Figure 5c. It can be seen from the inset that the Rb atoms are ordered and uniform in size and have no other coordinating atoms. This means that the crystal consists of Rb atoms [33]. Figure 5d shows the EDS elemental mapping analysis of 2 mol% Rb-ZnO/In_2_O_3_. This analysis shows that Zn (green) is distributed in the middle to form a block, and In (blue), O (red), and Rb (yellow) are distributed around ZnO. Where no other elements were detected, the corresponding elemental content estimation is shown in Figure 5e. This confirms that the prepared Rb-loaded ZnO/In_2_O_3_ composites consisted of In, O, Zn, and Rb. All of the above characterization results confirm the successful loading of Rb into the ZnO/In_2_O_3_ heterojunction composites. The dimensions of the ZnO/In_2_O_3_ heterojunction, as well as the heterostructure of ZnO/In_2_O_3_, were not changed before or after loading.

The X-ray photoelectron spectra (XPS) of ZnO/In_2_O_3_ and 2 mol% Rb-ZnO/In_2_O_3_ are shown in Figure S3. Figure S3a shows the full XPS spectra of ZnO/In_2_O_3_ and 2 mol% Rb-ZnO/In_2_O_3_, indicating the presence of zinc, indium, and oxygen. Figure S3b shows the Zn 2p spectra with two significant peaks at 1021.70 and 1044.68 eV, corresponding to Zn 2p_3/2_ and Zn 2p_1/2_, respectively, with a splitting value of about 23 eV. This confirms that the Zn atoms in all samples are in a fully oxidized state [34]. In Figure S3c, the In 3d spectra show peaks at 444.22 (In 3d_5/2_) and 451.72 eV (In 3d_3/2_) with a splitting value of about 7.5 eV, indicating the presence of In3^+^ [35]. X-ray photoelectron spectroscopy (XPS, Figure S3d) of the Rb 3d measurements shows that the binding energy at 103.6 eV belongs to the Rb 3d peak. Additionally, the peak for Rb 3d^+^ occurs at 102.3 eV, indicating that only a small fraction of the elemental Rb is oxidized [36]. The atomic percentages of ZnO/In_2_O_3_ and 2 mol% Rb-ZnO/In_2_O_3_ are shown in Table S1.

### 3.2. Gas-Sensing Performance

**Figure 6:**
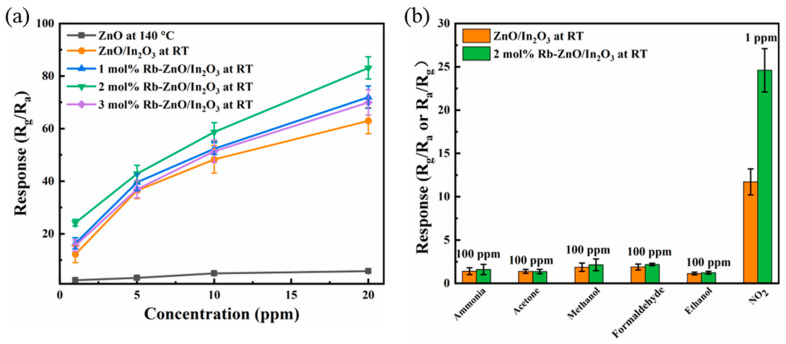
(**a**) Comparison of ZnO, ZnO/In_2_O_3,_ and 1–3 mol% Rb-ZnO/In_2_O_3_ sensor response values to 1–20 ppm NO_2_; (**b**) selectivity test of ZnO/In_2_O_3_ and 2 mol% Rb-ZnO/In_2_O_3_ sensor.

This work is dedicated to the development of high-performance, room-temperature sensors, so the operating temperature of the gas-sensing tests was uniformly chosen to be room temperature (24 °C). The response values of the ZnO at 140 °C and the ZnO/In_2_O_3_ heterojunction and 1–3 mol% Rb-loaded ZnO/In_2_O_3_ sensors at 1, 5, 10, and 20 ppm NO_2_ at room temperature were tested, as shown in Figure 6a. It can be seen that the response values of ZnO at an operating temperature of 140 °C were very low (four concentration response values: 1.55, 1.68, 1.82, and 1.93, respectively). Meanwhile, both the ZnO/In_2_O_3_ sensor and the Rb-ZnO/In_2_O_3_ sensor showed an enhanced effect on the detection of NO_2_. The response values of the pure ZnO/In_2_O_3_ sensor at four concentrations were 11.7, 37.1, 48.3, and 63, respectively; the response values of 1 mol% Rb-ZnO/In_2_O_3_ at four concentrations were 16.3, 39.6, 52.4, and 72, respectively; the response values of 2 mol% Rb-ZnO/In_2_O_3_ at four concentrations were 24.2, 42.9, 58.7, and 83.1; and the response values of 3 mol% Rb-ZnO/In_2_O_3_ at four concentrations were 15.56, 36.96, 51.37, and 70. The ZnO/In_2_O_3_ sensor not only achieved room-temperature detection of NO_2_ but also demonstrated a significantly improved response value compared to the ZnO sensor. The 2 mol% Rb-ZnO/In_2_O_3_ sensor achieved a higher response value for NO_2_ compared to the ZnO/In_2_O_3_ sensor, and at a low concentration of 1 ppm, the response value was approximately twice as high. The above experiments show that the Rb-loaded ZnO/In_2_O_3_ sensor is significantly superior to the ZnO and ZnO/In_2_O_3_ sensors. Meanwhile, the highest sensitivity to NO_2_ was achieved at 2 mol% Rb loading. Therefore, the subsequent gas sensitivity experiments were mainly investigated using the 2 mol% Rb-ZnO/In_2_O_3_ sensor. Then, the sensitivities of the ZnO/In_2_O_3_ and 2 mol% Rb-ZnO/In_2_O_3_ sensor were tested for 100 ppm of ammonia, acetone, methanol, formaldehyde, and ethanol and 1 ppm of NO_2_ gas, respectively, as shown in Figure 6b. The two sensors showed excellent gas sensitivity performance for the detection of 1 ppm of NO_2_ (the response value of the 2 mol% Rb-ZnO/In_2_O_3_ sensor was 24.2 and of pure the ZnO/In_2_O_3_ sensor was 11.7), which is clearly different from the other 100 ppm gases (the response values of both sensors were less than 2). To investigate the selectivity of the Rb-ZnO/In_2_O_3_ sensors, the selectivity coefficients were defined as S_A_/S_B_, where S_A_ and S_B_ are the values of the sensor response to NO_2_ gas and other gases, respectively [15]. The selectivity coefficients of the ZnO/In_2_O_3_ sensor for ethanol, formaldehyde, methanol, acetone, and ammonia were calculated to be 10.35, 6.22, 6.29, 8.48, and 8.36, respectively. Meanwhile, the selectivity coefficients of the 2 mol% Rb-ZnO/In_2_O_3_ sensor for ethanol, formaldehyde, methanol, acetone, and ammonia were 20.02, 11.28, 11.49, 18.08, and 15.37, respectively. These results indicate that the introduction of Rb improved the sensitivity and selectivity of the ZnO/In_2_O_3_ sensor.

**Figure 7:**
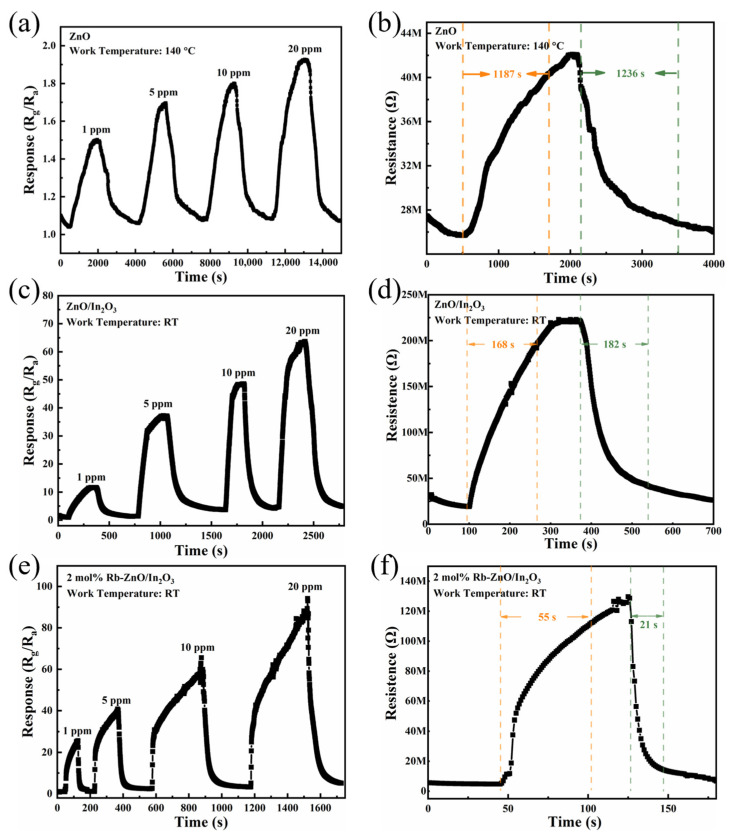
(**a**) Dynamic response–recovery curve versus different NO_2_ concentrations (1–20 ppm) of ZnO at 140 °C, (**b**) response–recovery time of the ZnO sensor to 1 ppm of NO2 at 140 °C, (**c**) dynamic response–recovery curve versus different NO_2_ concentrations (1–20 ppm) of ZnO/In_2_O_3_ at RT, (**d**) response–recovery time of the ZnO/In_2_O_3_ sensor to 1 ppm of NO_2_ at RT, (**e**) dynamic response–recovery curve versus different NO_2_ concentrations (1–20 ppm) of 2 mol% Rb-ZnO/In_2_O_3_ at RT, and (**f**) response–recovery time of the 2 mol% Rb-ZnO/In_2_O_3_ sensor to 1 ppm of NO_2_ at RT.

Subsequently, the response of the sensor was tested in the concentration range of 1–20 ppm, as was the transient response and recovery at 1 ppm. Figure 7a,b depict the dynamic response changes of the ZnO sensor when exposed to 1–20 ppm of NO_2_ at 140 °C and the response recovery curve at 1 ppm of NO_2_. The sensor exhibited poor NO_2_ response values of only 1.55 at 1 ppm and response and recovery times of 1187 and 1236 s, respectively. Therefore, the sensor’s performance for NO_2_ detection at a high temperature of 140 °C is limited. Figure 7c–f, to the contrary, shows the dynamic response changes of the pure ZnO/In_2_O_3_ and 2 mol% Rb-ZnO/In_2_O_3_ sensors when exposed to 1–20 ppm of NO_2_ at RT, as well as the response recovery curves at 1 ppm of NO_2_. The sensor response values for pure ZnO/In_2_O_3_ at 1, 5, 10, and 20 ppm of NO_2_ can be seen in Figure 7c. The resistance of the sensor was essentially stable at a baseline value when synthetic air was introduced, and the resistance showed an increasing trend when NO_2_ gas was introduced. Figure 7d showed the transient response curve of the pure ZnO/In_2_O_3_ sensor in a low-concentration 1 ppm of NO_2_ atmosphere, with response and recovery times of 168 and 182 s, respectively. Upon comparison, the ZnO/In_2_O_3_ sensor showed a substantial increase in the response value at high temperatures (from 1.55 to 11.7) and a reduction in the response and recovery times compared to ZnO.

**Figure 8:**
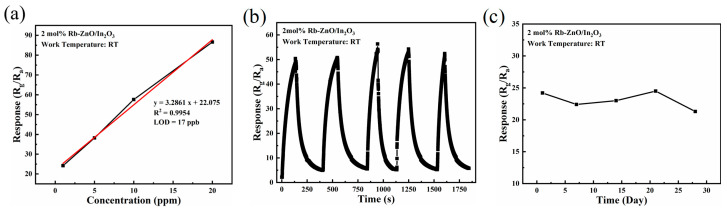
(**a**) Linear fit curve of the 2 mol% Rb-ZnO/In_2_O_3_ sensor to 1–20 ppm of NO_2_, (**b**) repeatability test of the 2 mol% Rb-ZnO/In_2_O_3_ sensor to 10 ppm of NO_2_, (**c**) long-term stability of the 2 mol% Rb-ZnO/In_2_O_3_ sensor to 1 ppm of NO_2_.

Figure 8a shows the linear fit curve of the 2 mol% Rb-ZnO/In_2_O_3_ sensor at an NO_2_ concentration of 1–20 ppm. The R^2^ value of the 2 mol% Rb-ZnO/In_2_O_3_ sensor was close to 1.0, indicating good linearity of the sensor in detecting NO_2_. This is important for practical applications requiring gas detection over a wide range. The calculated LOD for the 2 mol% Rb-ZnO/In_2_O_3_ sensor was 17 ppb, suggesting that this gas sensor can be used for NO_2_ detection at lower concentration levels. Figure 8b shows the repeatability test of the 2 mol% Rb-ZnO/In_2_O_3_ sensor at 10 ppm of NO_2_. After five repeated passes of 10 ppm of NO_2_ gas, the sensor showed a stable response and could return to the original baseline resistance. This means that the 2 mol% Rb-ZnO/In_2_O_3_ sensor has good repeatability. In addition, the long-term stability of the 2 mol% Rb-ZnO/In_2_O_3_ sensor for 10 ppm of NO_2_ is shown in Figure 8c. The response fluctuated slightly during the 28-day test period, demonstrating excellent long-term stability.

Table 1 shows a comparative analysis of the NO_2_ sensing performance of this sensor with other sensors documented in the literature. As illustrated in the figure, the Rb-ZnO/In_2_O_3_ sensor exhibited superior sensitivity compared to both the noble metal-modified ZnO and ZnO-based heterojunctions. Furthermore, in comparison to other sensors, the response value and response–recovery time of the 2 mol% Rb-ZnO/In_2_O_3_ sensor in this study were again enhanced. This may be attributed to the increase in active sites resulting from the loading of Rb and the rise in surface chemisorbed oxygen, which augments the redox rate. In conclusion, these findings offer novel insights into the potential for enhancing the NO_2_ sensing performance of ZnO-based sensors.

### 3.3. Gas-Sensing Mechanism

**Figure 9:**
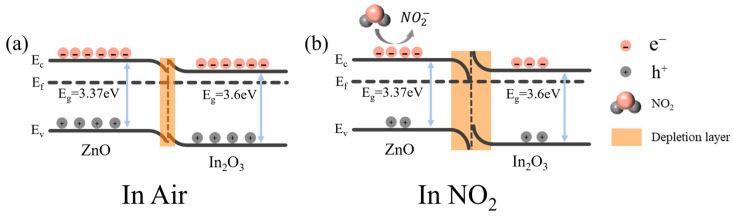
Schematic energy band structure of Rb-ZnO/In_2_O_3_ in (**a**) air and (**b**) NO_2_, where E_c_ is the conduction band, E_f_ is the Fermi level, E_v_ is the valence band, and E_g_ is the band gap.

Previous reports have generally used the surface depletion model to explain the sensing mechanism, which depends on the conductivity change of the sensing material during the adsorption and desorption of the target gas as shown in Figure 9 [43]. Oxygen is first adsorbed on the surface of ZnO/In_2_O_3_, then converted into chemisorbed oxygen species according to Equations (2) and (3) [1]. During this process, a large number of free electrons are trapped in their conduction band. As a result, a very thin electron depletion layer tends to form at the grain boundaries, as shown in Figure 9a. Subsequently, when Rb-ZnO/In_2_O_3_ is in a nitrogen dioxide atmosphere, due to the high electron affinity of nitrogen dioxide molecules, it can simultaneously trap free electrons from the conduction band of Rb-ZnO/In_2_O_3_ and react with chemisorbed oxygen species on the surface, as shown in Equations (4) and (5) [44]. In this process, the carrier concentration in the sensing material further decreases, the layer thickness increases further, and the conductivity decreases significantly, as shown in Figure 9b. Finally, the sensor returns to its original state after the nitrogen dioxide supply is turned off, as shown in Equation (6) [12,45].

(2) $$O_{2}\left(\mathrm{gas})\;\to O_{2}(\mathrm{ads}\right)$$

(3) $$O_{2}(\mathrm{ads})+e^{-}\to O_{2}^{-}(\mathrm{ads})$$

(4) $${NO}_{2}\left(gas\right)+e^{-}\to {NO}_{2}^{-}\left(ads\right)$$

(5) $${NO}_{2}\left(gas\right)+O_{2}^{-}+{2e}^{-}\to {NO}_{2}^{-}\left(ads\right)+{2O}^{-}$$

(6) $${NO}_{2}^{-}\left(ads\right)+{2O}^{-}\to {NO}_{2}\left(gas\right)+O_{2}+3e^{-}$$

It has been reported that Rb-loaded ZnO/In_2_O_3_ can improve the sensing performance of nitrogen dioxide gas at room temperature for two main reasons: first, the loaded Rb metal can act as a catalyst to promote chemical reactions of the target gas molecules and provide more active sites to enhance the adsorption and decomposition of these gas molecules [46,47]. Second, the loaded Rb metal can increase the electron transfer between ZnO/In_2_O_3_ and the gas and regulate the surface electron density of the ZnO/In_2_O_3_ material, thus improving the sensitivity and selectivity of the sensor [48]. Even though the above findings have been widely reported, the mechanism by which Rb loading influences the adsorption behavior of gas molecules on the surface of ZnO/In_2_O_3_ heterojunctions has not yet been thoroughly investigated. Therefore, we performed density functional theory-based calculations to reveal the effect of Rb-ZnO/In_2_O_3_ on NO_2_.

In the previously reported literature, the construction of heterojunctions with ZnO (002) and In_2_O_3_ (104) facets leads to a stable configuration [12]. Therefore, the (002) facet of the ZnO model and the (104) facet of the In_2_O_3_ model were investigated. In order to better match ZnO and In_2_O_3_ heterojunctions, single cell ZnO and In_2_O_3_ were expanded into 3 × 2 × 1 ZnO and 2 × 1 × 1 In_2_O_3_ supercells, respectively. 

**Figure 10:**
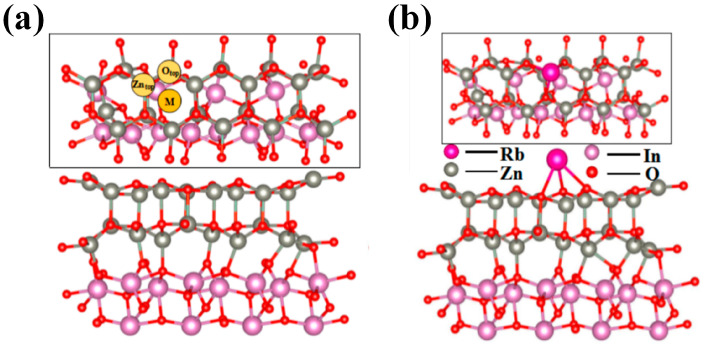
(**a**) Front and top views of the constructed ZnO/In_2_O_3_ heterostructure with three possible Rb adsorption sites; (**b**) front and top views of the structural model after adsorption of Rb atoms.

The degree of mismatch between the lattice parameters of the ZnO/In_2_O_3_ heterojunctions was calculated to be 1.09%, thus meeting the requirements for constructing heterostructure models (i.e., the mismatch is less than 5%). When the degree of mismatch was less than 5%, the constructed heterojunction was not susceptible to lattice mismatch [28,49]. The constructed ZnO/In_2_O_3_ was optimized, and the front and top views of the optimized structure are shown in Figure 10a. After structural optimization, a transition layer was formed at the interface, new In-O-Zn bonds appeared, and the structure did not collapse with broken bonds.

In order to construct the Rb-loaded ZnO/In_2_O_3_ model, three adsorption sites were selected for Rb-loaded adsorption. The stability of the Rb-loaded ZnO/In_2_O_3_ structure can be judged by calculating the binging energy (*E_b_*) as follows [50]:

*E_b_ = E_Rb-ZnO/In_*_2*O*3_ − *E_ZnO/In_*_2*O*3_ − *E_Rb_* (7)

where *E_Rb-ZnO__/In_*_2*O*3_ represents the total energy of the Rb-ZnO/In_2_O_3_ model, *E_ZnO/In_*_2*O*3_ represents the energy of the ZnO/In_2_O_3_ model, and *E_Rb_* represents the energy of the Rb monomer. The more negative the *E_b_*, the stronger the structure. According to Table 2, the binding energies were −0.094 eV for Zn_top_, −0.325 eV for O_top_, and −0.386 eV for M. In comparison, it was found that the binding energy of the overall system was the smallest when Rb was adsorbed on the M site of ZnO/In_2_O_3_. Therefore, the Rb-ZnO/In_2_O_3_ structure at the M adsorption site was the most stable, as shown in Figure 10b.

In order to better discuss the adsorption energy in the gas-sensitive mechanism analysis, the calculation of adsorption energy (*E_ads_*) is defined as follows [51]:

*E_ads_ = E_Total_ − E_Rb-ZnO/In*2*O*3*_ − E_Gas_* (8)

where *E_Total_*, *E_Rb-ZnO/In_*_2*O*3_, and *E_Gas_* are the total energy of the system, the energy of the Rb-ZnO/In_2_O_3_ model, and the energy of the gas molecules, respectively, and the magnitude of the adsorption energy represents the strength of the adsorption between the gas molecules and the gas-sensitive material.

In order to discuss the charge transfer quantity better in the gas-sensitive mechanism analysis, the charge transfer quantity (∆ρ) can be calculated and defined as follows [52]:

(9) $$∆\rho =\rho_{Total}-\rho_{Rb-ZnO/In2O3}-\rho_{Gas}$$

where $\rho_{Total}$, $\rho_{Rb-ZnO/In2O3}$, and $\rho_{Gas}$ are the total charge of the surface and adsorbed molecules, the charge of the Rb-ZnO/In_2_O_3_ model, and the charge of the molecules, respectively. The magnitude of the charge transfer represents the concentration of carriers between the gas molecules and the gas-sensitive material.

**Figure 11:**
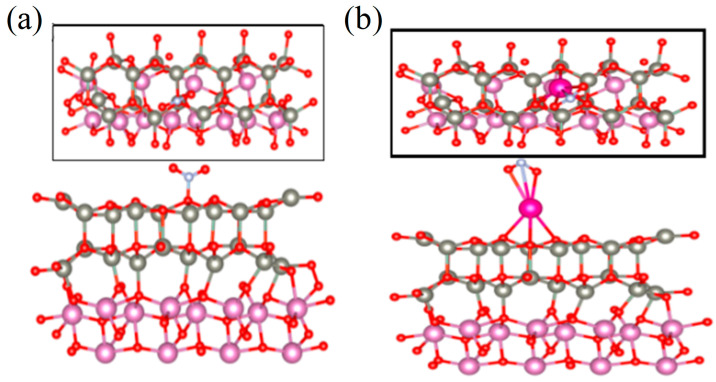
(**a**) Optimal configurations of the NO_2_ molecules adsorbed by (**a**) ZnO/In_2_O_3_ and (**b**) Rb-ZnO/In_2_O_3_.

Before calculating the adsorption energy and charge transfer, structural optimization is required. Figure 11 shows the optimal adsorption models of the ZnO/In_2_O_3_ structure and Rb-loaded ZnO/In_2_O_3_ structures for NO_2_. Figure S4 shows the optimal adsorption model of the ZnO/In_2_O_3_ and Rb-loaded ZnO/In_2_O_3_ structures for other gases. The calculated adsorption energies and charge transfers of the two structures for different gases are shown in Table 3.

**Figure 12:**
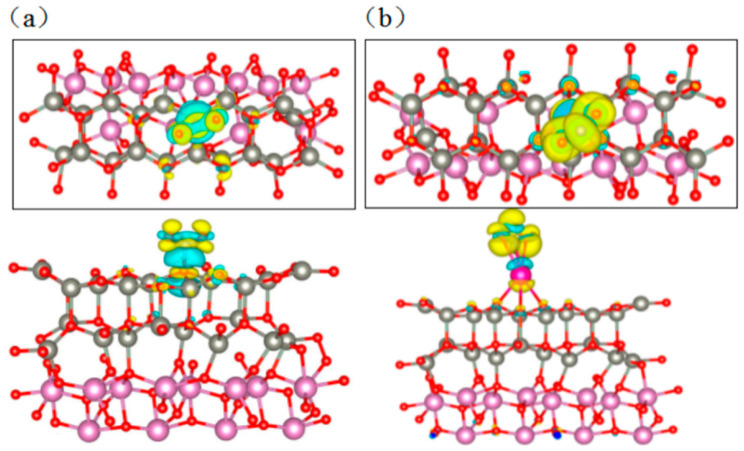
Differential charge density configurations of (**a**) NO_2_ adsorbed on ZnO/In_2_O_3_ and (**b**) Rb-ZnO/In_2_O_3_ (yellow areas represent electron accumulation, and blue areas represent electron depletion).

It can be seen from Table 3 that after NO_2_ gas adsorption on the ZnO/In_2_O_3_ structure, an N-O bond was formed between the gas molecule and the surface, with a bond length of 1.462 Å due to the adsorption effect. Compared to the adsorption of the other two gases, NO_2_ had the shortest bond length and the largest adsorption energy, indicating that ZnO/In_2_O_3_ has the best adsorption capacity for NO_2_ among the three gases. Meanwhile, the Rb-loaded ZnO/In_2_O_3_ structure also had the largest adsorption energy for NO_2_ among the three gases. From Figure 11b, it can be seen that Rb-N and Rb-O bonds were formed between the Rb-ZnO/In_2_O_3_ structure and NO_2_, and NO_2_ gas molecules adsorbed with Rb had a bond length of 2.339 Å, which is the shortest bond length compared to the other gases as well. This implies that the Rb-ZnO/In_2_O_3_ structure also had the best adsorption capacity for NO_2_. It is noteworthy that the adsorption energy of Rb-ZnO/In_2_O_3_ for NO_2_ was smaller than that of ZnO/In_2_O_3_ for NO_2_. This means that NO_2_ gas molecules are more easily desorbed on the surface of Rb-ZnO/In_2_O_3_, resulting in a faster recovery time of the Rb-ZnO/In_2_O_3_ sensor. This is consistent with the gas-sensitive test results observed previously (the recovery time of the Rb-loaded ZnO/In_2_O_3_ sensor was indeed shortened from 182 to 21 s). Interestingly, the charge transfer of NO_2_ adsorbed on Rb-ZnO/In_2_O_3_ was significantly larger than that of NO_2_ adsorbed on ZnO/In_2_O_3_. Figure 12 shows the charge difference plots of the adsorbed NO_2_ molecules for both structures, and the surface electrons of both structures were transferred toward NO_2_. However, it is obvious that the charge density of Rb-ZnO/In_2_O_3_ was much larger, and its charge transfer was 15 times that of ZnO/In_2_O_3_. The Rb-loaded metal increased the amount of electron transfer between the ZnO/In_2_O_3_ surface and NO_2_ gas, resulting in greater electron capture by NO_2_ gas from the ZnO/In_2_O_3_ surface. This means that the carrier concentration in the sensing material was further reduced, the depletion layer became thicker, and the conductivity decreased significantly, thus increasing the sensitivity and selectivity of the sensor. The above calculations fully illustrate the depth of the sensitization mechanism of the Rb-loaded ZnO/In_2_O_3_ sensor to NO_2_ gas.

## 4. Conclusions

In conclusion, pure and alkali metal Rb-loaded ZnO/In_2_O_3_ was successfully prepared using a combination of hydrothermal and impregnation methods. The experimental tests showed that the 2 mol% Rb-loaded ZnO/In_2_O_3_ sensor achieved an excellent response value of 24.2 to 1 ppm of NO_2_, with response and recovery times of 55 and 21 s, respectively. The response value, response time, and recovery time of the Rb-loaded ZnO/In_2_O_3_ sensor were significantly better than those of the pure ZnO and ZnO/In_2_O_3_ sensors. This indicates that Rb-loaded ZnO/In_2_O_3_ sensors have a more obvious performance enhancement for NO_2_.

The adsorption energies of NO_2_ with Rb-ZnO/In_2_O_3_ and ZnO/In_2_O_3_ and the charge transfer were calculated using DFT, and we found that NO_2_ molecules interacted more strongly with the former than the latter, while the degree of charge transfer was larger. These results suggest that the introduction of Rb plays an important role in increasing the adsorption capacity as well as the carrier concentration of the ZnO/In_2_O_3_ radical. In this work, the mechanism by which Rb loading enhances the NO_2_-sensing performance of ZnO/In_2_O_3_ heterojunctions was thoroughly explored through theoretical calculations and experimental validation, providing theoretical guidance and technical support for the development of high-performance room-temperature NO_2_ sensors. Future studies can further explore other rare metal loadings and their effects on the sensing performance of different gases, while the combination of advanced DFT computational simulation techniques will help to better understand the surface and interfacial properties of the materials, thus guiding the development and application of new sensing materials.

## Figures and Tables

**Table 1:** Comparison between the NO_2_ sensing performance of the 2 mol% Rb-ZnO/In_2_O_3_ gas sensor and other literature results.

Sensing Materials	Working Temperature	Concentration (ppm)	Response (R_g_/R_a_ or R_a_/R_g_)	Response–Recovery Time	Reference
Au-porous ZnO nanowires	RT	1	2.3	Not present	[37]
Pd-ZnO nanowires	100 °C	1	13.5	141/177 s	[38]
ZnSe/ZnO	200 °C	8	10.42	98/141 s	[39]
Pt-ZnO/PrGO	RT	5	1.76	528/702 s	[40]
In_2_O_3_/ZnO nanofibers	RT	1	6.0	36/68 s	[41]
ZnO/In_2_O_3_	RT	5	2.21	78/610 s	[42]
ZnO/In_2_O_3_	RT	10	29.1	61/39 s	[12]
2 mol% Rb-ZnO/In_2_O_3_	RT	1	24.2	55/21 s	This work

**Table 2:** Binding energy (*E_b_*) parameters of Rb at different adsorption sites of the ZnO/In_2_O_3_ model.

Structure Configurations	Adsorption Sites	*E_b_* (eV)
Rb-ZnO/In_2_O_3_	Zn_top_	−0.094
Rb-ZnO/In_2_O_3_	O_top_	−0.325
Rb-ZnO/In_2_O_3_	M	−0.386

**Table 3:** Adsorption energy (*E_ads_*), charge transfer (*Q*), and bonding distance (d) of ZnO/In_2_O_3_ and Rb-ZnO/In_2_O_3_ on different gas molecules.

Structure Configurations	Gas	*E_ads_* (eV)	*Q* (|*e*|)	d (Å)
ZnO/In_2_O_3_	NO_2_	−1.176	0.031	1.462
ZnO/In_2_O_3_	C_2_H_5_OH	−0.186	0.001	3.122
ZnO/In_2_O_3_	CH_3_COCH_3_	−0.319	0.026	2.523
Rb-ZnO/In_2_O_3_	NO_2_	−0.642	0.480	2.339
Rb-ZnO/In_2_O_3_	C_2_H_5_OH	−0.115	0.008	2.823
Rb-ZnO/In_2_O_3_	CH_3_COCH_3_	−0.278	0.005	2.837

## Data Availability

Data are available upon request.

## Supplementary Materials

The following supporting information can be downloaded at: https://www.mdpi.com/article/10.3390/s24165311/s1, Figure S1. SEM cross sections of sensing films; Figure S2. SEM images of (a) 1 mol% Rb-ZnO/In_2_O_3_, (b) 3 mol% Rb-ZnO/In_2_O_3_; Figure S3. XPS spectra of the obtained samples: (a) survey, (b) Zn 2p of ZnO/In_2_O_3_ and 2 mol% Rb-ZnO/In_2_O_3_, (c) In 3d of ZnO/In_2_O_3_ and 2 mol% Rb-ZnO/In_2_O_3_, (d) Rb 3d of 2 mol% Rb-ZnO/In_2_O_3_. Figure S4. Optimal configurations of C_2_H_5_OH molecules adsorbed by (a) ZnO/In_2_O_3_ and (b) Rb-ZnO/In_2_O_3_. Optimal configurations of CH_3_COCH_3_ molecules adsorbed by (c) ZnO/In_2_O_3_ and (d) Rb-ZnO/In_2_O_3_. Table S1. Elemental composition [at%] of the ZnO/In_2_O_3_ and 2 mol% Rb-ZnO/In_2_O_3_ powders, detected with XPS.
