# Supplementary material for: Enhanced NO2 Gas Sensing Properties Based on Rb-Doped ZnO/In2O3 Heterojunctions at Room Temperature: A Combined DFT and Experimental Study
Source: Sensors (Basel). 2024 Aug 16;24(16):5311. doi: 10.3390/s24165311 (PMC11359272; doi:10.3390/s24165311)
Supplement: Supplementary file 1 [file sensors-24-05311-s001.zip › sensors-3142321-supplementary.pdf]

# Supplementary Materials

## Enhanced $NO_2$ Gas Sensing Properties Based on Rb-Doped ZnO/$In_2O_3$ Heterojunctions at Room Temperature: A Combined DFT and Experimental Study

**Yaning Yang [1,*], Jiawen Cui [1], Zhihua Luo [1], Zhixin Luo [1] and Yanhui Sun [1,2]**

[1] College of Information &Communication Engineering, Dalian Minzu University, Dalian 116600, China
[2] School of Mechanical Engineering, Dalian University of Technology, Dalian 116024, China
* Correspondence: 20040662@dlnu.edu.cn

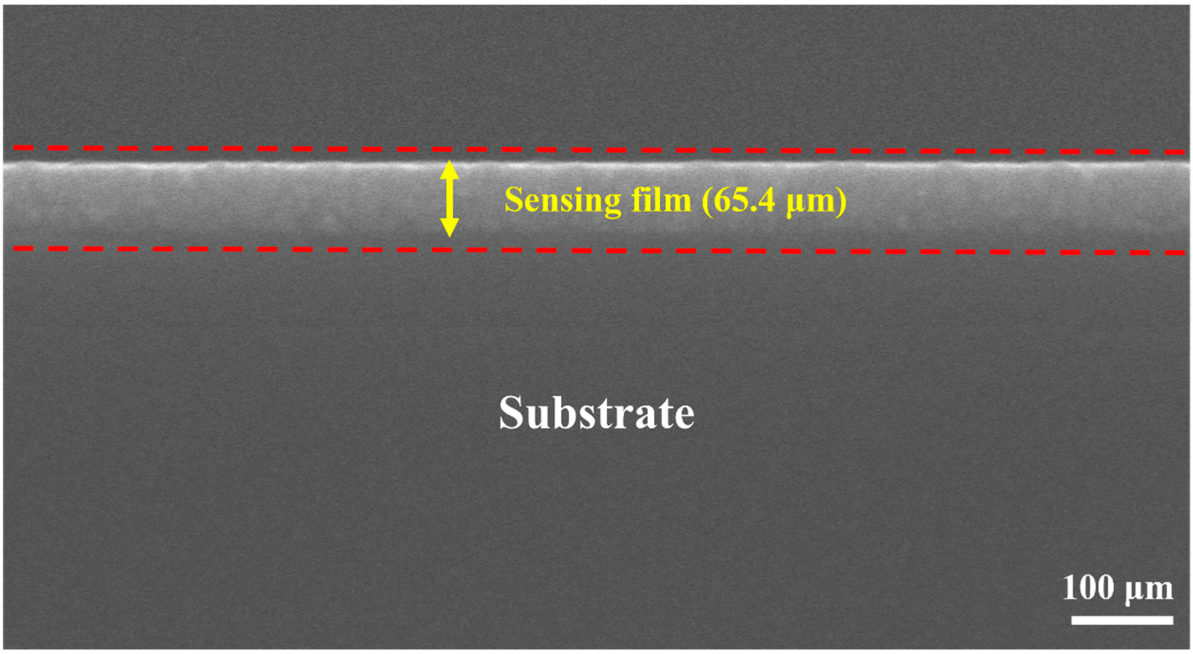


**Figure S1.** SEM cross sections of sensing films.

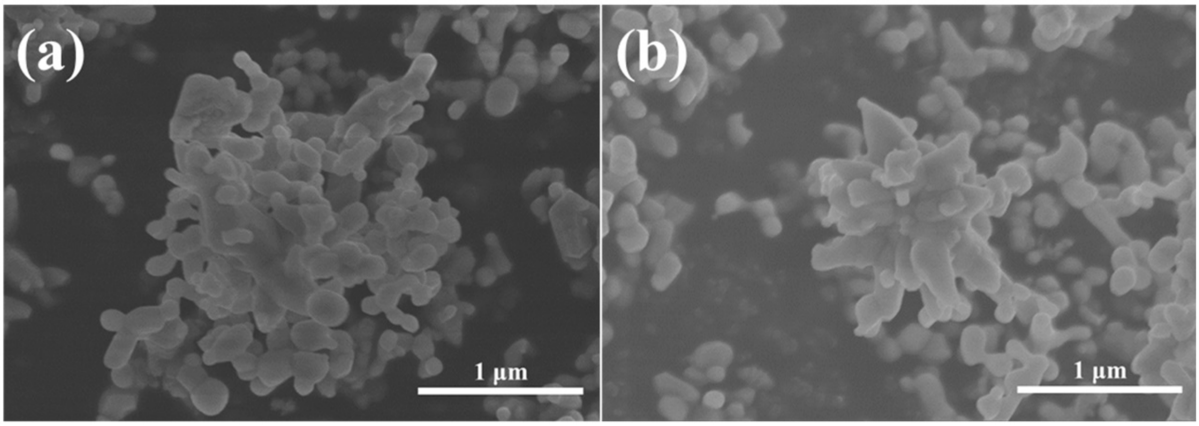


**Figure S2.** SEM images of (a) 1 mol% Rb−ZnO/$In_2O_3$, (b) 3 mol% Rb−ZnO/$In_2O_3$.

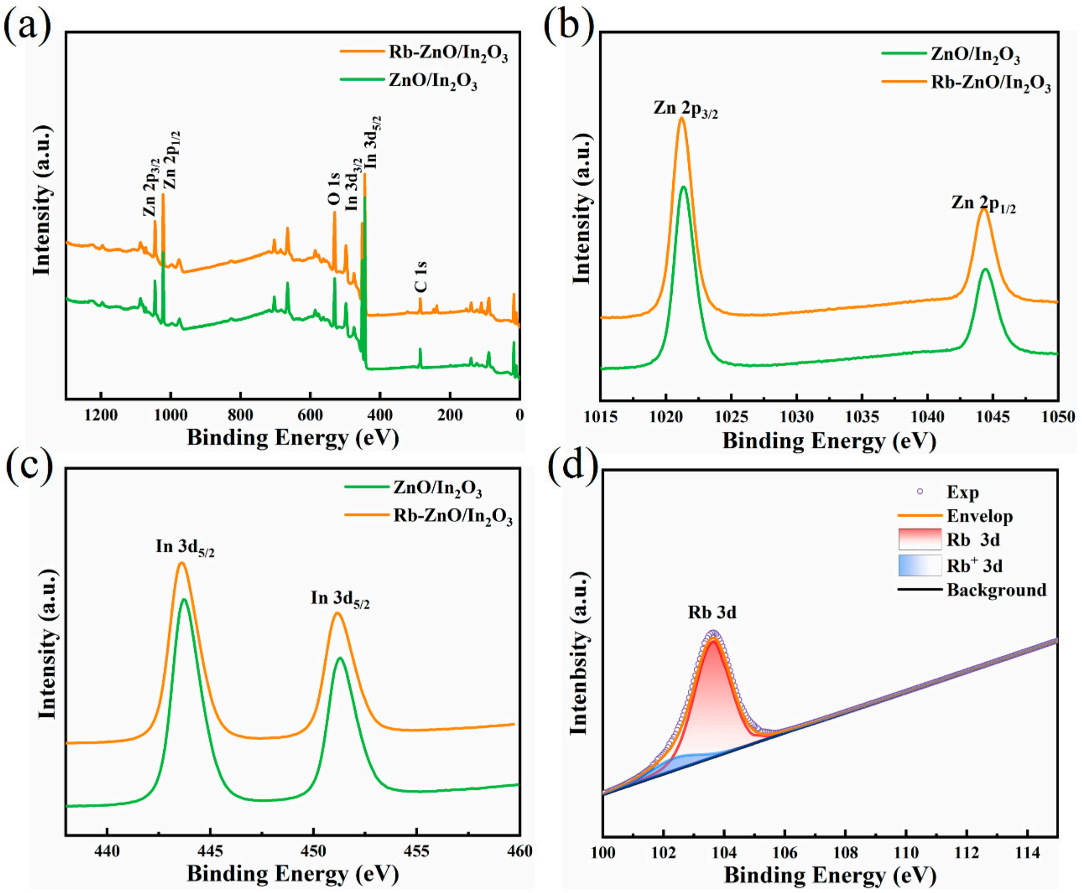


**Figure S3.** XPS spectra of the obtained samples: (a) survey, (b) Zn 2p of $ZnO/In_2O_3$ and 2 mol% Rb−$ZnO/In_2O_3$, (c) In 3d of $ZnO/In_2O_3$ and 2 mol% Rb−$ZnO/In_2O_3$, (d) Rb 3d of 2 mol% Rb−$ZnO/In_2O_3$.

**Table S1.** Elemental composition [at %] of the $ZnO/In_2O_3$ and 2 mol% Rb−$ZnO/In_2O_3$ powders, detected with XPS.

| Sample | O [% at] | Zn [% at] | In [% at] | Rb [% at] |
|---|---|---|---|---|
| $ZnO/In_2O_3$ | 49.82 | 25.57 | 24.61 | 0.0 |
| 2 mol% Rb−$ZnO/In_2O_3$ | 15.73 | 28.08 | 26.07 | 0.17 |

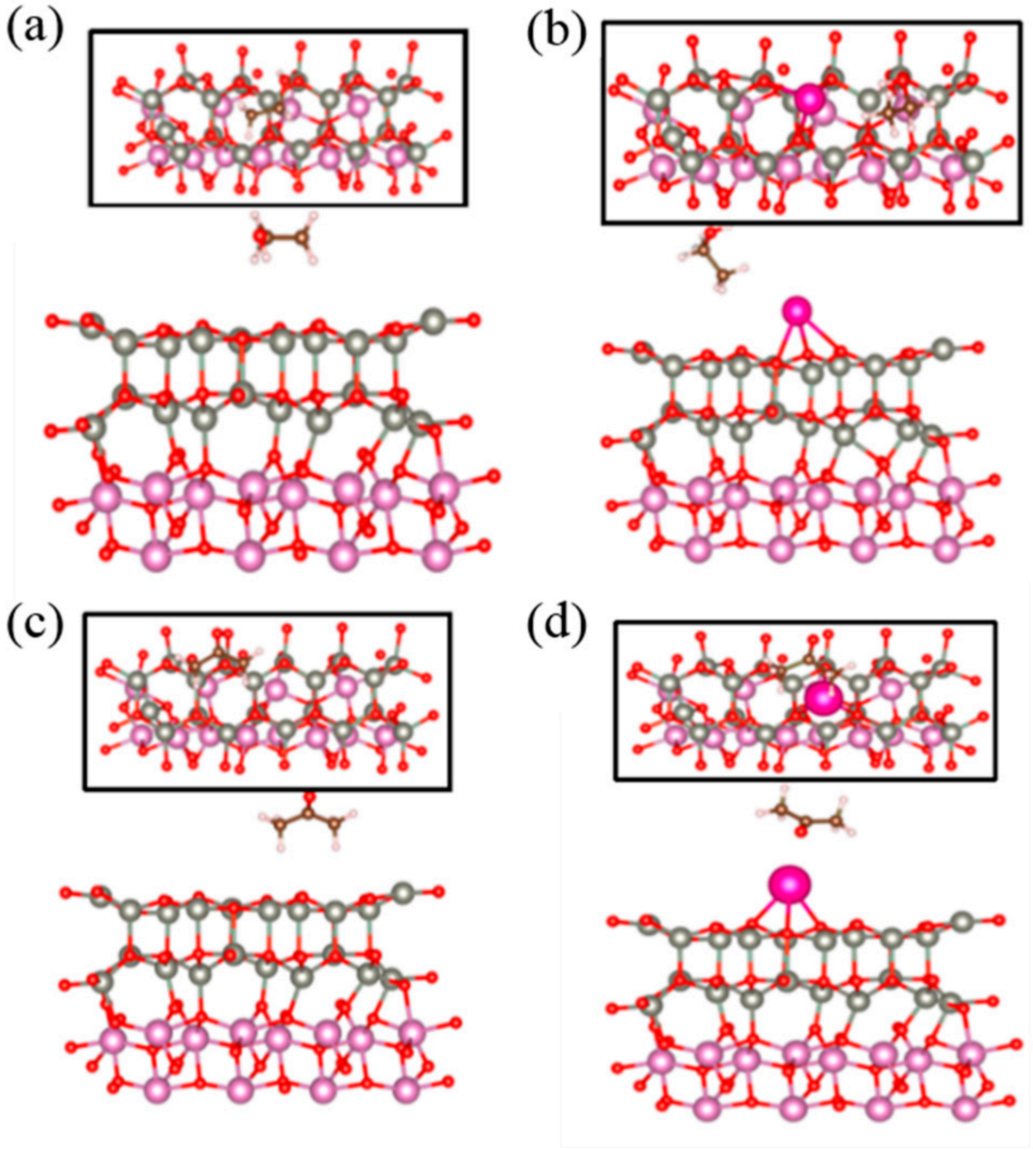


**Figure S4.** Optimal configurations of $C_2H_5OH$ molecules adsorbed by (a) $ZnO/In_2O_3$, and (b) Rb−$ZnO/In_2O_3$. Optimal configurations of $CH_3COCH_3$ molecules adsorbed by (c) $ZnO/In_2O_3$, and (d) Rb−$ZnO/In_2O_3$.
